# IFN-γ-STAT1-mediated CD8^+^ T-cell-neural stem cell cross talk controls astrogliogenesis after spinal cord injury

**DOI:** 10.1186/s41232-023-00263-9

**Published:** 2023-02-13

**Authors:** Jingyu Wang, Lintao Xu, Deqing Peng, Yongjian Zhu, Zhaowen Gu, Ying Yao, Heyangzi Li, Xi Cao, Chun-yan Fu, Mingzhi Zheng, Xinghui Song, Yueming Ding, Yueliang Shen, Jinjie Zhong, Ying-ying Chen, Jue Hu, Lin-lin Wang

**Affiliations:** 1 Department of Neurosurgery, Second Affiliated Hospital of Zhejiang University School of Medicine, Key Laboratory of Precise Treatment and Clinical Translational Research of Neurological Diseases, Hangzhou, 310009 China; 2 Department of Neurosurgery, Center for Rehabilitation Medicine, Zhejiang Provincial People’s Hospital (Affiliated People’s Hospital Hangzhou Medical College), Hangzhou, Zhejiang China; 3 Department of Neurointensive Care Unit, Second Affiliated Hospital of Zhejiang University School of Medicine, Hangzhou, 310009 China; 4 Department of Basic Medicine Sciences, Zhejiang University School of Medicine, Hangzhou, 310058 China; 5 School of Basic Medical Sciences & Forensic Medicine of Hangzhou Medical College, Hangzhou, 310053 China; 6 Central Laboratory, Women’s Hospital, Zhejiang University School of Medicine, Hangzhou, 310006 China; 7 School of Medicine, Zhejiang University City College, Hangzhou, 310015 China; 8 Department of Basic Medicine Sciences and Department of Obstetrics of the Second Affiliated Hospital, Zhejiang University School of Medicine, Hangzhou, 310058 China; 9 Department of Basic Medicine Sciences and Department of Orthopaedics of Sir Run Run Shaw Hospital, Zhejiang University School of Medicine, Hangzhou, 310058 China

**Keywords:** CD8 (+) T cells, Spinal cord injury, Neural stem cell, Interferon-γ, Astrocyte

## Abstract

**Background:**

Spinal cord injury (SCI) causes nearly all patients to suffer from protracted disabilities. An emerging therapeutic strategy involving the recruitment of endogenous neural stem cells (NSCs) has been developed. However, endogenous NSCs in the adult spinal cord differentiate into mostly astrocytes after traumatic injury, forming glial scars, which is a major cause of regeneration failure in SCI. Thus, understanding which factors drive the activation and differentiation of endogenous NSCs after SCI is critical for developing therapeutic drugs.

**Methods:**

The infiltration, state, and location of CD8^+^ T cells in spinal cord after traumatic injury were analyzed by flow cytometry and immunofluorescence (IF) staining. The Basso Mouse Scale (BMS) scores and rotarod testing were used for motor behavioral analysis. NSCs were co-cultured with CD8^+^ T cells. EdU assay was used to detect proliferative cells. Western blotting was used to analyze the expression levels of STAT1, p-STAT1, and p27. ChIP-seq and ChIP-qRT-PCR analyses were used to detect the downstream of STAT1. Nestin-CreERT2::Ai9 transgenic mice were used to genetic lineage tracing of Nestin^+^ NSCs after SCI in vivo.

**Results:**

A prolonged increase of activated CD8^+^ T cells occurs in the injured spinal cords. The behavioral analysis demonstrated that the administration of an anti-CD8 antibody promotes the recovery of locomotor function. Then, we discovered that CD8^+^ T cells suppressed the proliferation of NSCs and promoted the differentiation of NSCs into astrocytes by the IFN-γ-STAT1 pathway in vitro. ChIP-seq and ChIP-qRT-PCR analysis revealed that STAT1 could directly bind to the promoters of astrocyte marker genes GFAP and Aldh1l1. Genetic lineage tracing of Nestin^+^ NSCs demonstrated that most NSCs differentiated into astrocytes following SCI. Depleting CD8^+^ T cells reduced the differentiation of NSCs into astrocytes and instead promoted the differentiation of NSCs into oligodendrocytes.

**Conclusion:**

In conclusion, CD8^+^ T cells suppressed the proliferation of NSCs and promoted the differentiation of NSCs into astrocytes by the IFN-γ-STAT1-GFAP/Aldhl1l axis. Our study identifies INF-γ as a critical mediator of CD8^+^ T-cell-NSC cross talk and a potential node for therapeutic intervention in SCI.

**Supplementary Information:**

The online version contains supplementary material available at 10.1186/s41232-023-00263-9.

## Background

Spinal cord injury (SCI) always results in neurologic deficits and protracted disability, but the development of therapy in this area is still a daunting challenge [1–3]. Endogenous neural stem cells (NSCs) express classical stem cell markers (CD133, Sox2, Nestin, etc.), display critical stem cell properties (self-renewal and multipotentiality), and can replace lost cells and contribute to structural repair in SCI [4]. However, the activation of endogenous NSCs in mammals is insufficient for functional recovery following SCI, because most of them differentiate into astrocytes, forming an astrocyte scar post-CNS injury [5, 6]. In this context, it is imperative to understand which factors drive astrogliogenesis in SCI. It is reported that DNA demethylation and the JAK-STAT pathway collectively drive the onset of astrogliogenesis during CNS development [7–9]. NFIA gene is essential for astrogliogenesis, which can suppress oligodendrocyte differentiation by inhibiting the expression of Sox10 [10, 11]. In addition, overexpression of NFIB and Sox9 can efficiently induce astrocytes [12].

Grafting exogenous NSCs, which possess characteristics of integrating into injured sites and generating neuronal relays, could provide functional benefits for SCI [13]. However, it is difficult to obtain satisfying and safe exogenous NSCs for clinical treatment. Thus, activating endogenous NSCs to participate in neural repair is a more promising therapy for SCI. A recent study revealed that endogenous NSCs have the potential of generating remyelinating oligodendrocytes after SCI [14]. Recruitment of endogenous NSCs and inhibition of astrocyte differentiation appear to become primary aims for repairing CNS injury [15].

Neuroinflammation has been considered as a key pathological process for SCI. CD8^+^ T cells infiltrate into the parenchyma of the CNS in the subacute stage of injury and cause long-term neurological impairment [16–18]. The timing of NSC differentiation coincides with the onset of CD8^+^ T-cell infiltration following SCI [2, 19]. Importantly, a recent study has revealed that the infiltration of CD8^+^ T cells in old neurogenic niches suppresses the proliferation of NSCs, which indicated that CD8^+^ T cells may affect the fate of NSCs [20]. However, the relationship between CD8^+^ T cells and NSC differentiation after SCI remains poorly characterized.

Here, we found that increasing activated CD8^+^ T cells infiltrated the injured spinal cord over time. CD8^+^ T-cell depletion experiments using an anti-CD8 antibody and adoptive transfer of CD8^+^ T-cell experiments collectively demonstrated that CD8^+^ T cells worsen SCI. We further demonstrated that CD8^+^ T cells inhibited the proliferation of NSCs and promoted NSCs to differentiate into astrocytes by the IFN-γ-STAT1 pathway in vitro. We took advantage of Nestin-CreER^T2^::Ai9 mice that allow tamoxifen to permanently label NSCs and their progeny [21]. We found that NSCs were highly proliferating in the early stage of SCI. In the late stage, most of them turned into “quiescent” and differentiated into astrocytes, forming an astrocyte scar in the lesion rim. Depletion of CD8^+^ T cells promotes NSC proliferation, inhibits the differentiation of NSCs into astrocytes, but promotes the differentiation of NSCs into oligodendrocytes and white matter repair following SCI. Collectively, we conclude that CD8^+^ T cells promote NSCs to differentiate into astrocytes, which hinder tissue repair after SCI.

## Materials and methods

### Animals

Female C57BL/6 mice at age 10–12 weeks were used in all experiments in line with approved Institutional Animal Care and Use Committee protocols of Zhejiang University (no. ZJU20210236). All mice were maintained in the Laboratory Animal Center of Zhejiang University. Nestin-CreER^T2^, Rag2^−/−^, and Ai9 mice were obtained from the Jackson Laboratory. Nestin-CreER^T2^::Ai9 mice were used for lineage tracing Nestin^+^ NSCs as previously described [21]. Nestin-CreER^T2^::Ai9 mice were intraperitoneally injected with tamoxifen (150 mg/kg) once daily for 4 days to induce NSCs to express tdTomato (TdT) and left to rest for 2 weeks before surgery.

### Surgery and drug administration

The mice spinal cord contusion injury model was performed on 10- to 12-week-old mice as previously described [22]. Briefly, mice were deeply anesthetized with isoflurane inhalation. Then, a laminectomy was conducted at the T10 vertebral level. The contusive injury was performed using an NYU II impactor by rapidly dropping a 5 g rod onto the spinal cord from a height of 15 mm. The sham group mice underwent the same procedure without a contusion. All mice received a subcutaneous injection of flunixin (2.5 mg/kg) and ceftiofur sodium (5 mg/kg) once daily for 5 days after surgery. Bladders were carefully voided twice per day until the autonomic rhythm of the neurogenic bladder resumed. Rag2^−/−^ mice were intravenously injected by tail vein with CD8^+^ T cells (10^6 total cells) 3 days before SCI and every 3 days after SCI for adoptive cell therapy. To deplete CD8^+^ T cells, mice were intraperitoneally injected with a rat anti-mouse CD8 antibody (10 mg/kg, #BE0061, BioXCell) once daily for 3 days before SCI and repeated every 3 days after SCI. Control mice received equal rat IgG (#BE0090, BioXCell).

### Motor behavioral analysis

Before analysis, all mice were acclimated to the testing apparatus or open field for 1 h. Behavioral testing was performed via two observers blinded to the grouping and treatment.

The hind limb locomotor was assessed by the Basso Mouse Scale (BMS) scores at 0, 3, 7, 14, 21, and 35 days after SCI [23]. The hind limb locomotor activity was observed in an open field for 5 min at the same time on each test day (13:00). The initial score was similar for all mice. Mice with a difference of more than two score points between the left and right hind limbs were excluded.

Rotarod testing was used to measure coordination and gross motor capability. Mice were placed on an accelerating rotarod (from 0 to 40 rpm), and the latency to fall was recorded. All mice were subjected to one practice trial, followed by two test trials, with an interval of 20 min between trials.

### Tissue processing

For flow cytometry, mice were anesthesia and transcardially perfused with ice-cold saline to remove circulating cells. Spinal cord segments (8 mm) centered on the injured site were dissected and digested with 1 mg/ml type 2 collagenase (#17101015, Thermo Fisher Scientific) and 1 mg/ml papain (#76220, Sigma-Aldrich) in artificial cerebrospinal fluid (CSF) at 37 °C for 30 min, followed by trituration with 1-ml pipette. Subsequently, suspensions were washed twice with FACS buffer (PBS supplemented with 0.5% BSA), followed by density gradient centrifugation using 30%/70% Percoll (#17089109-1, GE Healthcare Life Science). Isolated single cells were collected from the 30 to 70% interface. Red blood cells (RBCs) were removed using an RBC lysis buffer (#420301, BioLegend).

For immunofluorescence, mice were perfused with ice-cold 4% paraformaldehyde (PFA) transcardially. The spinal cord segments were rapidly dissected and postfixed in 4% PFA overnight, followed by dehydration with 30% sucrose solution. OCT compound was used to embed all spinal cords. Then, spinal cords were sagittally or coronally cut into 10-μm-thick sections.

### Primary murine NSCs culture

Primary NSC culture was performed according to the previously published studies [24, 25]. The whole spinal cords derived from the embryos were dissected from E15 C57BL/6 mice in artificial CSF and digested with StemPro Accutase (#07922, STEMCELL Tech.) for 5 min at 37 °C. Following filtering through a 40-μm strainer and centrifugation, isolated cells at a concentration of 2 × 10^5^ cells/ml were cultured in 6-well plates in NeuraCult proliferation medium (#05702, STEMCELL Tech.) supplemented with 1% penicillin-streptomycin, 0.1% heparin solution (#07980, STEMCELL Tech.), 20 ng/ml EGF (#315-09, PeproTech), and 20 ng/ml bFGF (#450-33, PeproTech). NSCs were passaged every 3 days, and P2-4 NSCs were used for indicated experiments. For differentiation, NSCs at a concentration of 10^5^ cells/ml were seeded onto 6-well plates coated with 100 μg/ml poly-D-lysine (PDL, #P6407, Sigma-Aldrich) and cultured in NeuraCult differentiation medium (#05704, STEMCELL Tech.) for 7 days. To inhibit the expression of STAT1, the NSCs were transfected with STAT1 siRNA (50 μM) using INVI DNA/RNA Transfection Reagent (#IV1216100, Invigentech) for 24 h. The sequence of STAT1 siRNA was GCUGAACUAUAACUUGAAATT.

### *In vitro* CD8^+^ T-cell culture

Owing to little CD8^+^ T cells in healthy spinal cords, injured spinal cords derived from female adult C57BL/6 mice suffering from SCI for 14 days were dissociated into single-cell suspension as above described. The cells were washed by PBS twice and grown in complete medium, consisting of a 50/50 mixture of AIM-V (#12055091, Gibco) and RPMI medium (#22400105, Gibco) supplemented with 10% FBS (#10100147, Gibco), 1% penicillin-streptomycin (#15070063, Gibco), 1% GlutaMAX (#35050061, Gibco), 25 mM HEPES (#15630080, Gibco), and IL-2 (600 IU/ml, #212-12, PeproTech). The T cells were rapidly expanded by anti-CD3/CD28 microbeads (#11456D, Gibco) for 2 days. After 2 weeks, CD8^+^ T cells were sorted using CD8 Microbeads (#130-116-478, Miltenyi Biotec.). The cell purity of CD8^+^ T cells was more than 95% as confirmed by flow cytometry (data not shown). To generate conditioned media (CM), CD8^+^ T cells (10^5/ml) were grown in NeuraCult proliferation or differentiation media supplemented with 600 IU/ml IL-2 and anti-CD3/CD28 microbeads for 24 h. To neutralize IFN-γ, an anti-IFN-γ antibody (10 μg/ml, #BE0055, BioXCell) was added to the culture medium of CD8^+^ T cells.

### EdU proliferation assay

The proliferation assay of NSCs was performed with an EdU Proliferation Kit (#ab219801, abcam) according to the manufacturer’s instruction. NSCs were treated with CM for 48 h. After 48 h, NSCs were incubated with 10 μM Edu for 3 h, followed by digestion with StemPro Accutase, fixation with 4% PFA, and permeabilization with 0.5% Triton X-100. Then, NSCs were incubated with iFluor 488 to label EdU for 30 min. EdU-positive cells, defined as proliferating phase cells, were analyzed with a flow cytometer.

### Flow cytometry

Cells were collected and washed with FACS buffer twice, followed by incubation with anti-mouse CD16/32 (Fc blocker, #553141, BD Biosciences) for 5 min at 4 °C. Then, cells were stained with anti-mouse CD3e (APC, #553066, BD Biosciences), CD4 (PE, #553652, BD Biosciences), CD8 (BV605, #100744, BioLegend), CD69 (PECY7, #104511, BioLegend), CTLA4 (PE, #553720, BD Biosciences), PD-1 (FITC, #135213, BioLegend), CD8 (FITC, #100705, BioLegend), CD4 (APCCY7, # 100413, BioLegend), or CD8 (PECY7, #100721, BioLegend) for 30 min at 4 °C. Each sample was washed with FACS buffer twice and resuspended in 0.5 ml FACS buffer. DAPI was used to exclude dead cells. Fluorescent cells were detected with CytoFLEX LX Flow Cytometer (Beckman).

### Immunofluorescence (IF) staining

Cells and spinal cord sections were fixed with 4% PFA, followed by blocking with 5% normal donkey serum (#017-000-121, Jackson ImmunoResearch) and 0.2% Triton X-100 in PBS for 1 h at room temperature. Then, they were stained with rabbit anti-CD8 (1:500, #ab217344, abcam); rabbit anti-Ki67 (1:200, #MA5-14520, Thermo Fisher Scientific); goat anti-CD31 (1:200, #AF3628, Novus); goat anti-Sox2 (1:200, #AF2018, Novus); rabbit anti-Sox10 (1:500, #ab227680, abcam); goat anti-Olig2 (1:200, #AF2418, Novus); rabbit anti-Neun (1:500, #ab177487, abcam); rabbit anti-MBP (1:500, #ab218011, abcam); chicken anti-GFAP (1:2000, #ab4674, abcam); rabbit anti-RFP (1:200, #600-406-379, Rockland); chicken anti-Map 2 (1:2000, #ab5392, abcam); chicken anti-Nestin (1:200, #NB100-1604, Novus); goat anti-choline acetyltransferase (ChAT, 1:200, #NBP1-30052, Novus); and chicken ani-RFP (1:200, #600-906-379, Rockland) at 4 °C overnight. Subsequently, cells and sections were washed with PBS three times for 10 min and incubated with secondary antibodies (1:1000) for 2 h and 1 μg/ml DAPI for 5 min at room temperature. All secondary antibodies were obtained from Jackson ImmunoResearch as follows: Alexa Fluor (AF) 488-Donkey Anti-Goat IgG (#705-546-147; AF594-Donkey Anti-Rabbit IgG (#711-586-152); AF594-Donkey Anti-Chicken IgY (#703-585-155); AF594-Donkey Anti-Goat IgG (#705-586-147); AF488-Donkey Anti-Rabbit IgG (#711-546-152); Cy5-Donkey Anti-Goat IgG (#705-175-147); and AF488-Donkey Anti-Chicken IgY (#703-546-155). Cells and sections were washed with PBS three times for 10 min and visualized with an Olympus FV3000 confocal microscope.

### Enzyme-linked immunosorbent assay (ELISA)

Spinal cord segments (8 mm) were collected at 0, 3, 7, 14, and 35 dpi after SCI and homogenized in lysis buffer at 4 °C. Protein concentrations were determined using a BCA protein assay kit. Then, tissue lysates were diluted to a final concentration of 1 mg/ml. An ELISA kit (#ELM-IFNg-1, RayBiotech) was used to detect the release of IFN-γ after SCI in accordance with the manufacturer’s protocol.

### Western blot analysis

NSCs were collected and dissolved in RIPA lysis buffer supplemented with protease/phosphatase inhibitors. Protein concentrations were determined using a BCA protein assay kit and adjusted to 3 mg/mL. Proteins were separated using sodium dodecyl sulfate-polyacrylamide gel electrophoresis and transferred onto nitrocellulose membranes. The membranes were blocked in 5% BSA and incubated with primary antibodies at 4 °C overnight. The primary antibodies included the following: rabbit anti-STAT1 (1:1000, #14994, CST); rabbit anti-pSTAT1 (Y^701^, 1:1000, #9167, CST); rabbit anti-P27 (1:1000, #3686, CST); and mouse anti-GAPDH (1:5000, #6004-1-lg, Proteintech). Three times after washes with TBS-T buffer, the membranes were incubated with anti-mouse IgG (DyLight™ 800 Conjugate, #5257, CST) and anti-rabbit IgG (DyLight™ 680 Conjugate, #5366, CST) for 1 h at room temperature. Three times after washes with TBS-T buffer, immunoblot bands were visualized by an Odyssey infrared imaging system (LI-COR Biosciences).

### Quantitative RT-PCR (qRT-PCR)

Total RNA was extracted from NSCs using TRIzol (#15596018, Thermo Fisher Scientific). A PrimScript RT Reagent Kit with gDNA Eraser (#RR047A, Takara) was used for cDNA synthesis. All primers were shown in Supplementary Table S1. RT-PCR was conducted using TB Green Premix Ex Taq II (#RR820, Takara) on LightCycler 480 II system (Roche). Data were collected and analyzed by the LightCycler 480 software (1.5.0).

### Chromatin immunoprecipitation (ChIP) and ChIP-seq

NSCs were treated with CM derived from CD8^+^ T-cell culture for 24 h and then collected for ChIP. ChIP was performed using a SimpleChIP Enzymatic Chromatin IP Kit (Magnetic Beads, #9003, CST) in accordance with the manufacturer’s instructions. Briefly, the NSCs were fixed with 1% formaldehyde at room temperature for 10 min followed by incubation with glycine at a final concentration of 0.125 M to quit the cross-linking reactions. Then, the NSCs were lysed and digested with micrococcal nuclease to cut the genomic DNA into 150–900 bp fragments. Input DNA samples were incubated with proteinase K and RNase at 65 °C for de-cross-linking followed by purification. DNA fragments of interest were isolated using 20 μg of an anti-STAT1 antibody (no. 14994, CST) or rabbit IgG (#2729, CST) with ChIP-grade protein G Magnetic Beads. Then, the DNA fragments were de-cross-linked and purified for quantitative RT-PCR and sequencing. All primers were shown in Supplementary Table S2.

The ChIP and Input DNAs were performed with the standard consecutive enzymatic steps of end repairing, adding one “A” nucleotide, and ligating barcoded adaptor, followed by PCR and size selection of 150–350 bp for DNA libraries preparation. The DNA libraries were sequenced on Illumina Nova 6000. Reads were filtered using Fastp V0.20 and aligned to the mouse genome (mm10) using the Bowtie algorithm. Duplicate reads, uniquely mapped reads (mapping quality < = 15), reads containing more than five nucleobases “N,” and reads less than 36 bp were removed. Peak locations were defined using the MACS algorithm (V2.1.1) with a cutoff of *p*-value = 1e-5. Known false ChIP-seq peaks were removed. Peaks were visualized using IGV software.

### Statistical analysis

The data were analyzed using GraphPad Prim (8.0). Student’s *t*-tests were used when comparing two groups. Multiple groups were analyzed by one-way ANOVA with Tukey’s multiple comparisons posttest or two-way ANOVA-RM with Bonferroni’s post hoc correction. The data were presented as the mean ± standard error of the mean (SEM). *P* < 0.05 was regarded as statistical significance.

## Results

### Infiltration of CD8^+^ T cells increased in the injured spinal cord

**Fig. 1:**
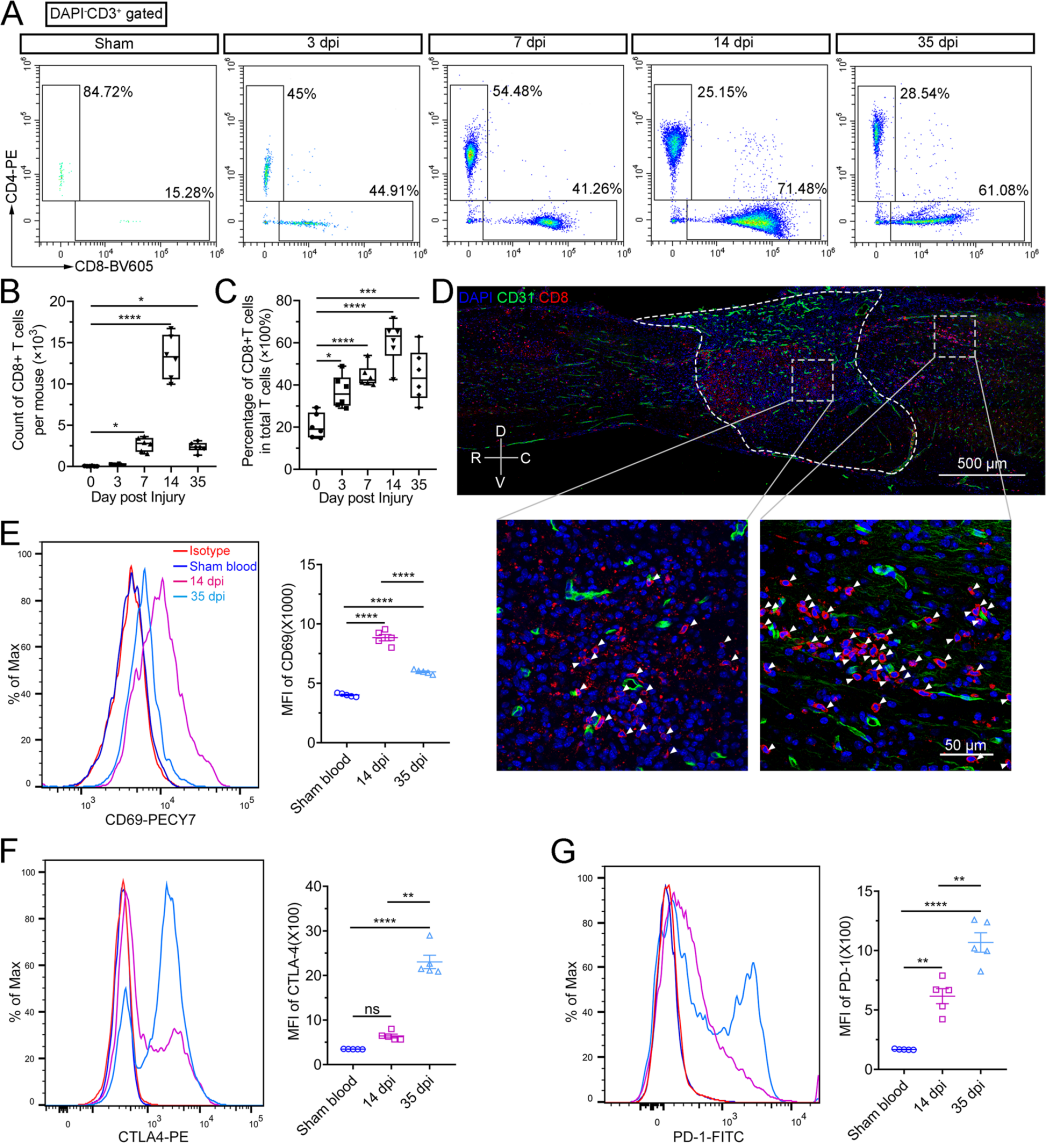
Infiltration of activated CD8^+^ T cells increased in the injured spinal cords. **A** Representative flow cytometry images showing the percentage of CD8^+^ T cells (CD3^+^CD4^−^CD8^+^) at 0, 3, 7, 14, and 35 dpi after SCI, *n* = 6 independent mice. **B** and **C** Statistical box plots of **A**, *P < 0.05, ***P < 0.001, ****P < 0.0001, one-way ANOVA with Tukey. **D** Immunofluorescence images of a sagittal section showing the location of CD8^+^ T cells (red) after SCI at 14 dpi. CD31 (green) is used to label endothelial cells of blood vessels. The lesion border was outlined by a dashed white line. V, ventral; D, dorsal; R, rostral; C, caudal. Scale bar, 500 μm or 50 μm. **E**–**G** Flow cytometry images showing the expression levels of CD69, CTLA-4, and PD-1 in CD8^+^ T cells in sham blood, 14 dpi, and 35 dpi groups, respectively. *n* = 5 independent mice. **P < 0.01, ****P < 0.0001, one-way ANOVA with Tukey. MFI, mean fluorescence intensity

We explored dynamic changes in the CD8^+^ T-cell (CD3^+^CD4^−^CD8^+^) population in the spinal cord in the acute, subacute, and chronic stages of traumatic injury (3, 7, 14, and 35 dpi) using flow cytometry (Fig. 1A and Supplementary Fig. 1A). The total numbers of infiltrating CD8^+^ T cells increased after SCI, peaked at 14 dpi (13.28 ± 1.08), and fell at 35 dpi (2.34 ± 0.24) (Fig. 1B). The proportion of CD8^+^ T cells in total T cells was also increased in the injured spinal cords, reaching a peak of 57.53% ± 3.8% at 14 dpi (Fig. 1C). Immunofluorescence staining showed that CD8^+^ T cells, which were invisible in healthy spinal cords (Supplementary Fig. 1B), were widely localized within the lesion core and rim of the extravascular parenchyma after SCI (Fig. 1D). The higher CD69 (an early T-cell activation marker) expression of CD8^+^ T cells at 14 dpi (Fig. 1E and Supplementary Fig. 1C) compared with other time points indicated that the increased CD8^+^ cells in the subacute stage of SCI were mainly an activated phenotype. However, the enhanced expression of T-cell exhaustion markers (CTLA-4 and PD-1) in CD8^+^ T cells at 35 dpi might account for the decreased number of CD8^+^ T cells in the chronic stage of traumatic injury (Fig. 1 F–G and Supplementary Fig. 1D).

**Fig. 2:**
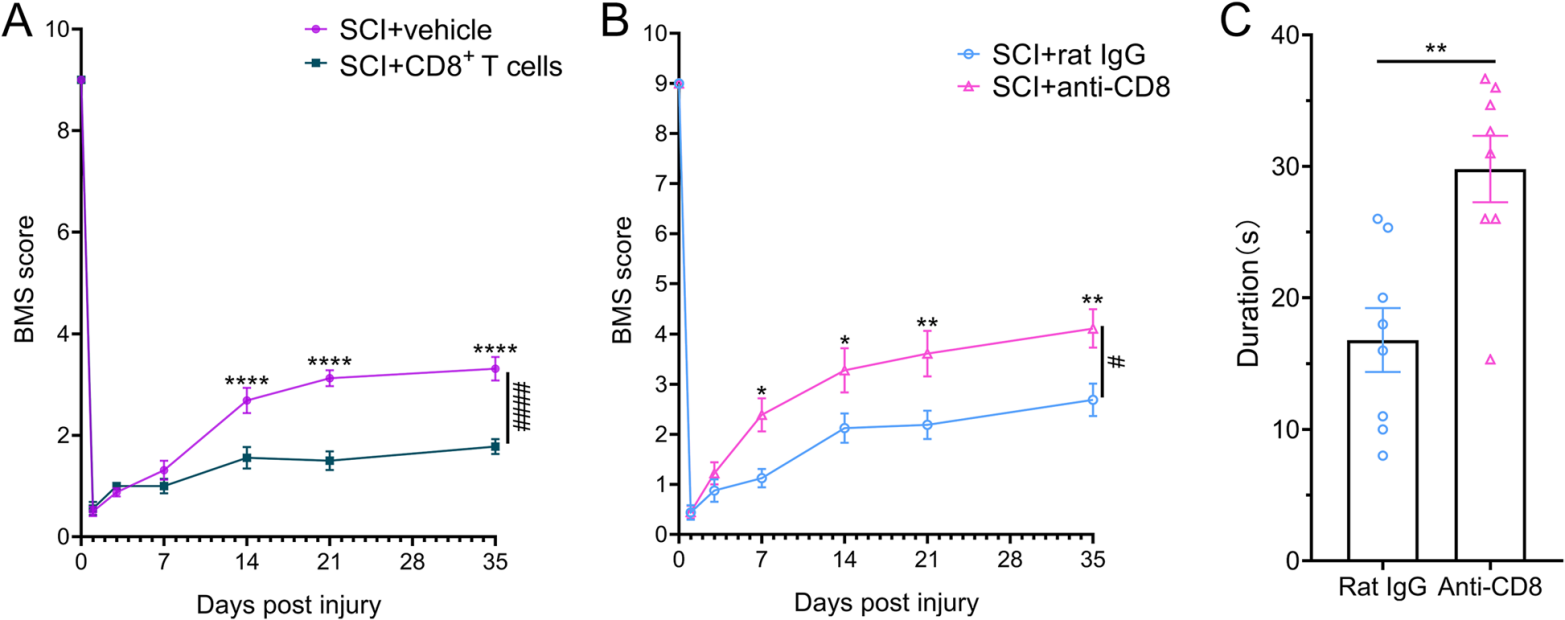
Infiltration of CD8^+^ T cells hindered the locomotor functional recovery of SCI. **A** BMS score showing locomotor function in Rag2^−/−^ mice with adoptive CD8^+^ T-cell therapy after SCI. *n* = 8 independent mice. ****P < 0.0001, ####*P* < 0.0001, two-way ANOVA-RM with Bonferroni. **B** BMS scores showing locomotor function in wild-type mice with the administration of anti-CD8 antibody after SCI. *n* = 8 independent mice. *P < 0.05, **P < 0.01, #P < 0.05, two-way ANOVA-RM with Bonferroni. **C** Rotarod assays showing ameliorative locomotor functional recovery after administration of anti-CD8 antibody at 35 dpi. *n* = 8 independent mice. **P < 0.01, Student’s *T*-test

To explore the effect of CD8^+^ T-cell infiltration on the recovery of locomotor function in SCI mice, CD8^+^ T cells were adoptively transferred into mature-T-cell-deficient Rag2^−/−^ transgenic mice. Transplantation of CD8^+^ T cells increased the number of CD8^+^ T cells in the spinal cord and deteriorated locomotor functional recovery of SCI mice (Fig. 2A and Supplementary Fig. 2A). Depleting CD8^+^ T cells by using an anti-CD8 antibody potentiated locomotor functional recovery of SCI mice (Fig. 2 B–C and Supplementary Fig. 2B). These results revealed that infiltration of CD8^+^ T cells in the spinal cords hindered the recovery of SCI.

### CD8^+^ T cells suppressed the proliferation of NSCs via the IFN-γ-STAT1 pathway *in vitro*

**Fig. 3:**
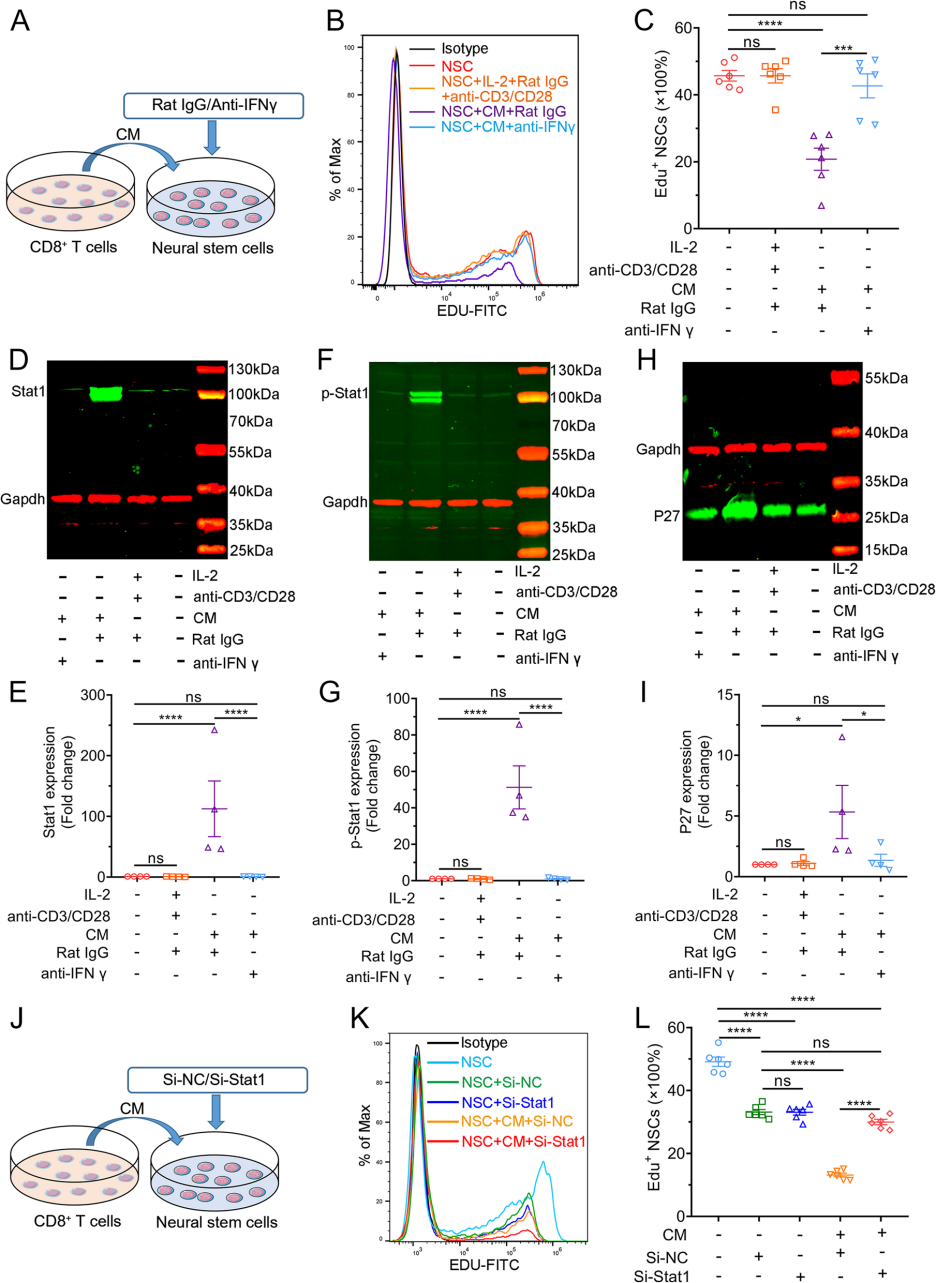
CD8^+^ T cells inhibit NSC proliferation via the IFN-γ-Stat1 pathway in vitro. **A** Experimental design, CM: conditioned medium. **B** Flow cytometry showing EdU-positive NSCs (proliferative NSCs) with CD8^+^ T-cell-derived conditioned medium with or without an anti-IFN-γ antibody. **C** Statistical graph of **B**. *n* = 6. ***P < 0.001, ****P < 0.0001, one-way ANOVA with Tukey. **D**–**I** Western blotting showing the expression levels of Stat1, p-Stat1 (Tyr705), and P27 in NSCs. *n* = 4. *P < 0.05, ****P < 0.0001, one-way ANOVA with Tukey. **J** Experimental design, CM, conditioned medium. **K** Flow cytometry showing EdU-positive NSCs with CD8^+^ T-cell-derived conditioned medium with or without Stat1 knockdown. **L** A statistical graph of **K**. *n* = 6. ****P < 0.0001, one-way ANOVA with Tukey

At first, we investigated whether CD8^+^ T cells could affect the proliferation of NSCs. Immunofluorescence staining showed that NSCs (Nestin^+^, Sox2^+^) were successfully isolated from the spinal cord and could differentiate into neurons, astrocytes, or oligodendrocytes (Supplementary Fig. 3). CD8^+^ T cells were cultured to obtain a conditioned medium (CM). ELISA analysis showed that many IFN-γ was secreted by CD8^+^ T cells in CM (Supplementary Fig. 4). Flow cytometry indicated that the percentage of EdU^+^ NSCs (proliferative NSCs) was more than twofold lower in CM treatment (20.76% ± 3.32%) compared with control (45.7% ± 1.61%), whereas other compounds (IL-2, anti-CD3/CD28 microbeads, and rat IgG) had no obvious effects (Fig. 3 A and B). An antibody that neutralizes murine IFN-γ inhibited CM-induced NSC proliferation (Fig. 3 A–C). Only CM induced the expression of STAT1 and p-STAT1 (Y^701^), which are the downstream molecules of the IFN-γ signal pathway, in NSCs (Fig. 3D-G). Moreover, CM increased the expression of P27, a cell cycle inhibitor, which was reversed by an anti-IFN-γ antibody (Fig. 3 H and I). To further explore whether the inhibiting effects of CD8^+^ T cells on the proliferation of NSCs are dependent on STAT1, STAT1 was downregulated by siRNA (Supplementary Fig. 5). The decrease in the proliferation of NSCs in the presence of CM was reversed by knocking down Stat1 (13.15% ± 0.59% versus 29.98% ± 0.85%) (Fig. 3 J–L). Taken together, these results demonstrated that CD8^+^ T cells inhibited the proliferation of NSCs via the IFN-γ-STAT1 pathway in vitro.

### CD8^+^ T cells promoted NSCs to differentiate into astrocytes *in vitro*

**Fig. 4:**
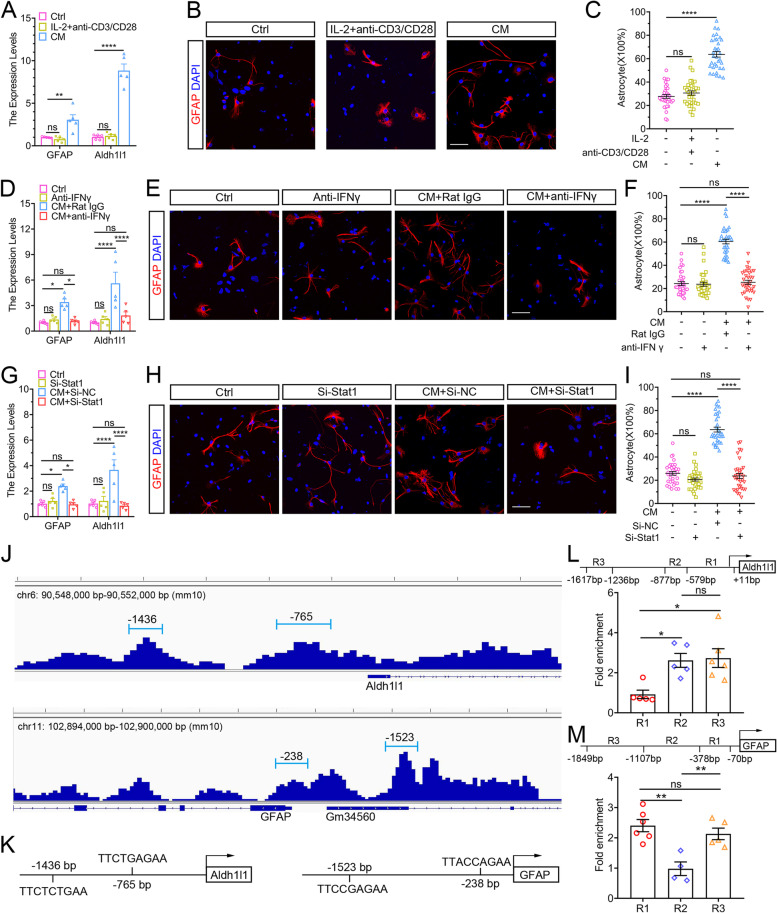
CD8^+^ T cells promoted the differentiation of NSCs into astrocytes via the IFN-γ-Stat1 pathway in vitro. **A** qRT-PCR showing the expression of astrocyte markers (GFAP and Aldh1l1) in NSCs with or without a conditioned medium for 7 days. *n* = 5. **P < 0.01, ****P < 0.0001, one-way ANOVA with Tukey. **B** and **C** Immunofluorescence showing the percentage of astrocytes (GFAP^+^ cells) differentiated from NSCs with or without conditioned medium for 7 days. *n* = 35. ****P < 0.0001, one-way ANOVA with Tukey. Scale bar: 50 μm. **D** qRT-PCR showing the expression levels of GFAP and Aldh1l1 in NSCs with or without an anti-IFN-γ antibody for 7 days. *n* = 5. *P < 0.05, ****P < 0.0001, one-way ANOVA with Tukey. **E** and **F** Immunofluorescence staining showing the percentage of astrocytes differentiated from NSCs with or without an anti-IFN-γ antibody for 7 days. *n* = 35. ****P < 0.0001, one-way ANOVA with Tukey. Scale bar, 50 μm. **G** qRT-PCR showing the expression levels of GFAP and Aldh1l1 in NSCs with or without Stat1 siRNA for 7 days. *n* = 5. *P < 0.05, ****P < 0.0001, one-way ANOVA with Tukey. **H** and **I** Immunofluorescence staining showing the percentage of astrocytes differentiated from NSCs with or without Stat1 siRNA for 7 days. *n* = 35. ****P < 0.0001, one-way ANOVA with Tukey. Scale bar, 50 μm. **J** Visual representation of Stat1 ChIP-seq enrichment in the vicinity of the Aldh1l1 and GFAP locus using IGV software (V2.13.2). **K** Stat1 binding sites in the vicinity of the Aldh1l1 and GFAP locus. **L** Stat1 ChIP-qRT-PCR showing specific enrichment of the R2 and R3 regions upstream of the Aldh1l1 gene. R1 and R2: *n* = 5, R3: *n* = 6, *P < 0.05, one-way ANOVA with Tukey. **M** Stat1 ChIP-qRT-PCR showing specific enrichment of the R1 and R3 regions upstream of the GFAP gene. R1: *n* = 6, R2: *n* = 4, R3: *n* = 5, **P < 0.01, one-way ANOVA with Tukey

We next explored whether CD8^+^ T cells affect the differentiation of NSCs. qPCR results revealed that the expression of astrocyte markers (GFAP and Aldh1a1) was upregulated in NSCs treated with CD8^+^ T-cell-derived CM (Fig. 4A). Immunofluorescence staining of GFAP^+^ astrocytes showed the similar result that the percentage of astrocytes differentiated from NSCs was significantly higher in the CM-treated group than in the control group (63.67% ± 2.33% versus 27.67% ± 1.65%) (Fig. 4 B and C). Instead, CM inhibited NSCs to differentiate into oligodendrocytes and neurons (Supplementary Fig. 6A). Then, we further investigated whether these effects rely on the classic cytokine secreted by CD8^+^ T cells: IFN-γ, as they do in the proliferation property of NSCs. Blocking IFN-γ reversed the effects of CD8^+^ T cells on the differentiation of NSCs (Fig. 4 D–F and Supplementary Fig. 6B). Likewise, the activation and accumulation of STAT1 are essential for the effects of CD8^+^ T cells on the differentiation of NSCs (Fig. 4 G–I and Supplementary Fig. 6C). These results suggested that CD8^+^ T cells could promote NSCs to differentiate into astrocytes via the IFN-γ-Stat1 pathway in vitro.

To identify the target genes of transcript factor STAT1, ChIP-seq was performed using chromatin harvested from NSCs. The results demonstrated that two peaks of Stat1 enriched reads binding to both promoter regions of Aldh1l1 (−765 bp and −1436 bp) and GFAP (−238 bp and −1523 bp) genes (Fig. 4J). And specific binding elements of STAT1 do exist at these peak locations (Fig. 4K). Furthermore, qRT-PCR revealed the binding sites of endogenous STAT1 were found at two upstream regions (R2 and R3) of Aldh1l1 and two upstream regions (R1 and R3) of GFAP, respectively (Fig. 4 L and M). These results revealed that STAT1 could directly boost the expression levels of astrocyte markers GFAP and Aldh1l1 by binding their promoters in NSCs.

### CD8^+^ T cells suppressed the proliferation of NSCs *in vivo*

**Fig. 5:**
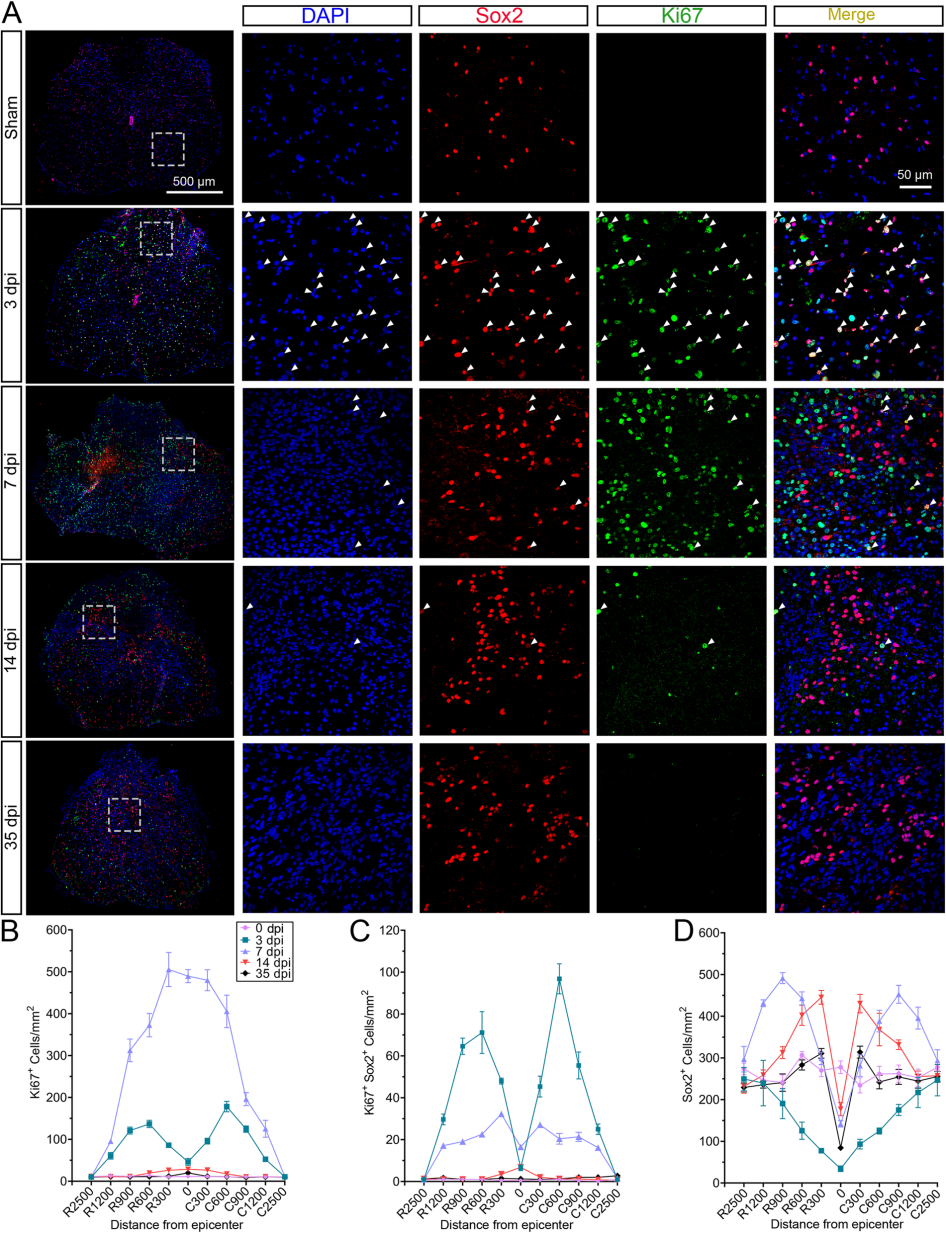
NSCs extensively proliferated within the lesion rim after SCI in mice. **A** Immunofluorescence staining showing the spatiotemporal distribution of NSCs (Sox2^+^, red) and proliferating cells (Ki67^+^, green) in the sham group, as well as within the lesion rim at 3, 7, 14, and 35 dpi. *n* = 4. Scale bar: 500 μm or 50 μm. **B** Quantification of the total number of proliferative cells per mm^2^ of tissue. **C** Quantification of the total number of proliferative NSCs per mm^2^ of tissue. **D** Quantification of the total number of NSCs per mm^2^ of tissue

The dynamic changes in the population of NSCs and their proliferation property following SCI remain unknown. Here, we found that SCI caused the hyperproliferative status of NSCs. More proliferative cells (Ki67^+^ cells) were discovered at 7 dpi than that at other time points (Fig. 5 A and B). However, only approximately 0.5 NSCs/mm^2^ were proliferating in the healthy spinal cords (Fig. 5C). Proliferative NSCs (Ki67^+^Sox2^+^) were rapidly increased within the lesion rim at 3 dpi and decreased over time (Fig. 5C). The total numbers of NSCs (Sox2^+^) were greatly decreased at the lesion epicenter in the acute stage, while it was increased within the lesion rim at 7 and 14 dpi after SCI (Fig. 5D).

**Fig. 6:**
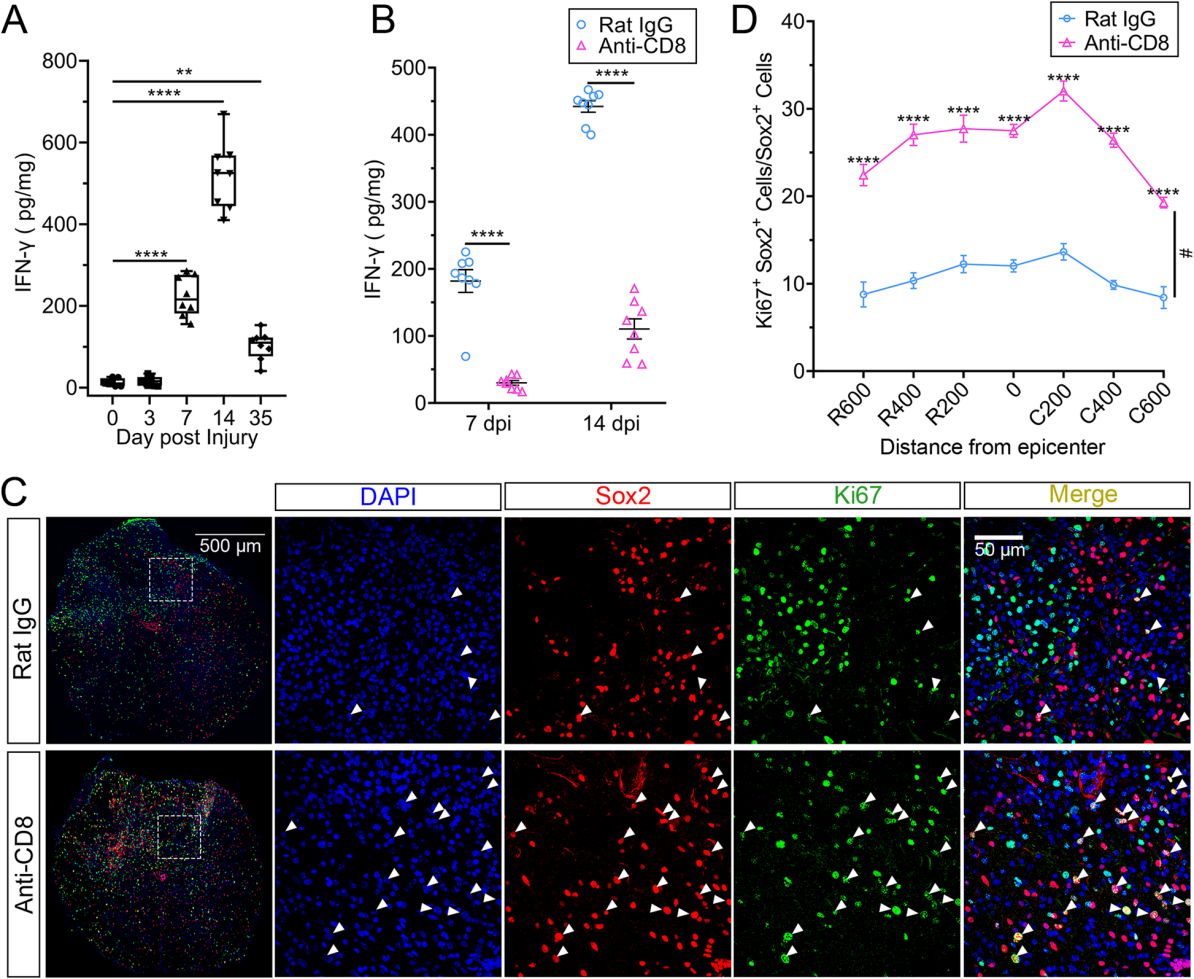
CD8^+^ T cells inhibited NSC proliferation in vivo. **A** Elisa analysis showing the level of IFN-γ at 0, 3, 7, 14, and 35 dpi after SCI, *n* = 8, **P < 0.01, ****P < 0.0001, one-way ANOVA with Tukey. **B** Elisa analysis showing the level of IFN-γ after the administration of an anti-CD8 antibody at 7 and 14 dpi. Equal rat IgG was used for control, *n* = 8, ****P < 0.0001, one-way ANOVA with Tukey. **C** Immunofluorescence staining showing the change of proliferating NSCs with or without the administration of an anti-CD8 antibody after SCI at 7 dpi. Scale bar, 500 μm or 50 μm. **D** A statistical graph of **C**. *n* = 4. ****P < 0.0001, #*P* < 0.05, two-way ANOVA-RM with Bonferroni

The timing of the decrease in the number of proliferative NSCs coincides with the onset of CD8^+^ T-cell infiltration. We next assessed whether CD8^+^ T cells could also inhibit the proliferation of NSCs in vivo. ELISA analysis revealed that the level of IFN-γ increased after SCI, peaked at 14 dpi, and fell at 35 dpi, whose trend was similar to the dynamic change of the total numbers of CD8^+^ T cells (Fig. 6A). Depleting CD8^+^ T cells using an anti-CD8 antibody resulted in a substantial decrease in the content of IFN-γ, revealing that IFN-γ was mainly produced and released by CD8^+^ T cells after SCI (Fig. 6B). Proliferative NSCs were increased after the administration of an anti-CD8 antibody (Fig. 6 C and D). In sight of these results, we concluded that CD8^+^ T cells could suppress the proliferation of NSCs in vivo.

### CD8^+^ T cells promoted NSCs to differentiate into astrocytes *in vivo*

**Fig. 7:**
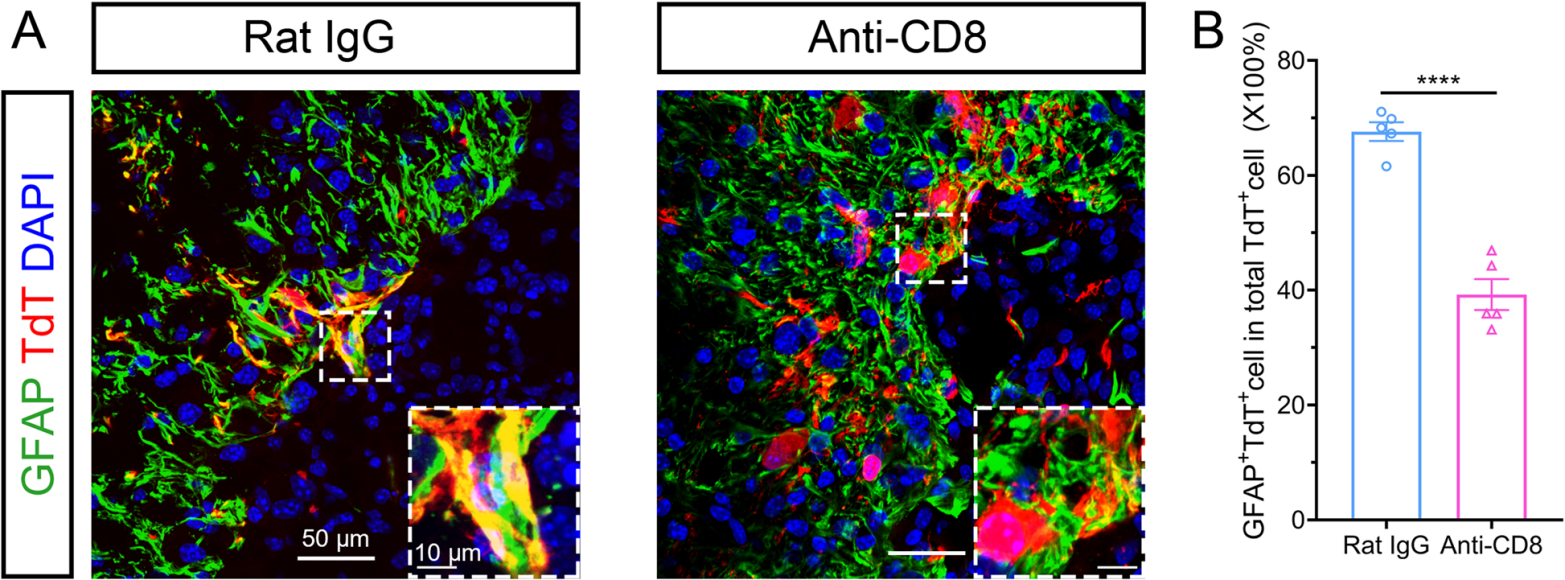
CD8^+^ T cells promoted NSCs to differentiate into astrocytes in vivo. **A** Immunofluorescence staining showing the percentage of GFAP^+^ astrocytes differentiated from NSCs in lineage tracing mice (Nestin-CreERT2::Ai9 mice) after the administration of an anti-CD8 antibody at 14 dpi. Equal rat IgG was used for control. Scale bar, 50 μm or 10 μm. **B** A statistical graph of **A**, *n* = 5, ****P < 0.0001, Student’s *t*-test

Eventually, we explored the effects of CD8^+^ T cells on NSC differentiation in vivo. Nestin-CreERT2::Ai9 mice were used for lineage tracing NSCs after SCI. NSCs and their progeny were permanently labeled with TdT (red fluorescence) after tamoxifen treatment. Approximately 70% of NSCs differentiated into astrocytes after SCI at 14 dpi, which was significantly inhibited when depleting CD8^+^ T cells (Fig. 7). Endogenous NSCs have been reported to have the potential of generating remyelinating oligodendrocytes after SCI [14]. However, our study showed that less than 3% of NSCs differentiated into oligodendrocytes (Sox10^+^ TdT^+^) in the SCI mice. Depleting CD8^+^ T cells caused a significant increase in oligodendrocyte differentiation (Supplementary Fig. 7A). And myelin sheath labeled by MBP was markedly increased after depleting CD8^+^ T cells (Supplementary Fig. 7B). Due to the motor functional recovery after depleting CD8^+^ T cells, we further asked whether ChAT^+^ motor neurons were myelinated by NSC-derived oligodendrocytes. IF staining showed that ChAT^+^ motor neuronal axon can be surrounded by NSC-derived myelin sheath (TdT^+^, MBP^+^) (Supplementary Fig. 7C). Notably, endogenous NSCs could not differentiate into neurons after SCI whether CD8^+^ T cells were infiltrated or not in the spinal cord (Supplementary Fig. 7D). These results demonstrated that CD8^+^ T cells promoted NSCs to differentiate into astrocytes in vivo.

## Discussion

It has been reported that some patients with SCI suffer from progressive deterioration of neurological functions for which chronic inflammation is considered a key factor [26, 27]. Lymphocytes are regarded to play a vital role in chronic inflammation during SCI [28]. But the recognition of the role of CD8^+^ T cells in SCI is limited. Our study indicates that activated CD8^+^ T cells infiltrate the spinal cord parenchyma for a long time after traumatic injury. CD8^+^ T cells promote NSCs to differentiate into astrocytes by the IFN-γ-Stat1 axis, which hinders the functional recovery of SCI. Depleting CD8^+^ T cells inhibits NSCs to differentiate into astrocytes, which favors locomotor functional recovery.

We demonstrated that CD8^+^ T cell is the main lymphocyte subtype in SCI. Microglia can form a protective scar that facilitates inflammatory containment after SCI [29–31]. However, CD8^+^ T cells are not restricted in the lesion core by the glial scar, the reason for which should be further explored. Generally, IFN-γ is secreted by Th1, Th17.1, CD8^+^ T cells, and NK cells in many physiological and pathological conditions. However, it is primarily produced by CD8^+^ T cells in the traumatic spinal cord, which might be because the number of CD8^+^ T cells infiltrated in the spinal cord was higher than other immune cells that produce IFN-γ after SCI [32]. Furthermore, IFN-γ is a key driver of the expression of PD-L1 that triggers T-cell exhaustion [33]. We found that CD8^+^ T cells were reduced in the late stage of SCI, which could be a reason for the increased expression of exhaustion markers such as PD-1 and CTLA-4 in these T cells. In addition, is IFN-γ the only mediator for CD8^+^ T-cell-NSC cross talk? Our results showed that IFN-γ neutralization completely reversed the effects of CD8+ T cell on NSCs’ proliferation and differentiation, which indicated that IFN-γ is required for CD8^+^ T-cell-NSC cross talk

On the other hand, an emerging therapeutic strategy involving the recruitment of endogenous NSCs, which rely on their cardinal properties (self-renewal and multipotency), has been developed [4]. However, endogenous NSCs in the spinal cord display non-multipotency, which is associated with the microenvironment within the spinal cord [34, 35]. Endogenous NSCs could be activated and solely generate gliogenic progeny following SCI, producing mostly astrocytes that form glial scars [36]. In this context, approximately 95% of the NSC progeny differentiates into astrocytes, and 5% differentiates into oligodendrocytes, though NSCs have the potential capability of differentiating into oligodendrocytes [14, 37]. Thus, understanding which factors determine the activation and differentiation of endogenous NSCs following SCI is critical for developing therapeutic drugs. In this study, we identify INF-γ as a critical mediator of CD8^+^ T-cell-NSC cross talk following SCI. Inhibition of CD8^+^ T cells boosts the proliferation of NSCs, generating enough NSCs to repair injured tissue and replace the lost cells. Importantly, inhibition of CD8^+^ T cells reduces astrocyte differentiation and instead promotes oligodendrocyte differentiation. Notably, our results also showed that inhibition of CD8^+^ T cells promotes oligodendrocyte differentiation, which may due to the balance between the astrocyte and oligodendrocyte differentiation from gliogenic stem cells after SCI. And the detailed mechanism should be further explored. However, inhibition of CD8^+^ T cells does not reverse the aneurogenic phenotype of NSCs following SCI, which may be due to the inaccessibility of promoters of the neurogenic transcription factors in spinal cord NSCs [14]. Thus, we raised the possibility that targeting CD8^+^ T cells may help in white matter repair and functional recovery.

There are also some hurdles in our study. It is unclear whether CD8^+^ T cells worsen SCI exclusively by affecting NSCs, so the utilization of NSCs depleting mice in our future study will continue to address this issue. On the other hand, direct inhibition of CD8^+^ T cells causes systemic immunosuppression and neoplasia. We speculated that blocking IFN-γ is probably safer, which is required to be further explored in the future. In addition, Stat1 may exist independently of IFN-γ for CD8^+^ T-cell-NSC cross talk. Thus, the link between Stat1 and IFN-γ needs to be further explored. Finally, the NSCs used in vitro are taken from the spinal cord of fetal mice, whereas spinal cord NSCs from adult mice may be originally preferred.

## Conclusions

In conclusion, CD8^+^ T cells suppressed the proliferation of NSCs and promoted the differentiation of NSCs into astrocytes by the IFN-γ-STAT1-GFAP/Aldhl1l axis.

## Supplementary Information

**Additional file 1: Table S1.** Primers. **Table S2.** Primers.

**Additional file 2: Supplementary Figure 1.** Infiltration of increasing activated CD8+ T cells after SCI. **Supplementary Figure 2.** Flow cytometry analysis showed the efficiency of transplantation or depletion (B) of CD8+ T cells in spinal cord of SCI mice. **Supplementary Figure 3.** Identification and differentiation of cultured NSCs. **Supplementary Figure 4.** ELISA showed the expression of IFN-γ in conditioned medium. **Supplementary Figure 5.** Western blotting showed the expression of Stat1 in NSCs with or without Stat1 siRNA. **Supplementary Figure 6.** CD8+ T cells inhibited the differentiation of NSCs into oligodendrocytes and neurons. **Supplementary Figure 7.** Depleting CD8+ T cells promotes white matter repair after SCI.

## Data Availability

All data related to this paper can be requested from the corresponding author.

## Abbreviations

SCI: Spinal cord injury
NSCs: Neural stem cells
CM: Conditioned medium
BMS: Basso Mouse Scale
RBCs: Red blood cells
IF: Immunofluorescence
ChAT: Choline acetyltransferase
AF: Alexa Fluor
ELISA: Enzyme-linked immunosorbent assay
ChIP: Chromatin immunoprecipitation
