# Supplementary material for: IFN-γ-STAT1-mediated CD8+ T-cell-neural stem cell cross talk controls astrogliogenesis after spinal cord injury
Source: Inflamm Regen. 2023 Feb 13;43:12. doi: 10.1186/s41232-023-00263-9 (PMC9926765; doi:10.1186/s41232-023-00263-9)
Supplement: Supplementary file 1 — Additional file 1: Table S1. Primers. Table S2. Primers. [file 41232_2023_263_MOESM1_ESM.docx]

Table S1.

| **Gene** | **Forward primer** | **Reverse primer** |
| --- | --- | --- |
| GFAP | CCCTGGCTCGTGTGGATTT | GACCGATACCACTCCTCTGTC |
| Aldh1l1 | CAGGAGGTTTACTGCCAGCTA | CACGTTGAGTTCTGCACCCA |
| Olig2 | TCCCCAGAACCCGATGATCTT | CGTGGACGAGGACACAGTC |
| Ptplb | ACGGCGTACCTGGTCATCTA | CTATGGTAGCTCCCCTTAGCC |
| Tubb3 | TAGACCCCAGCGGCAACTAT | GTTCCAGGTTCCAAGTCCACC |
| Neun | ATCGTAGAGGGACGGAAAATTGA | GTTCCCAGGCTTCTTATTGGTC |

Table S2.

| **Regions** | **Forward primer** | **Reverse primer** |
| --- | --- | --- |
| **Aldh1l1** |  |  |
| R1 | GCATAAAGCCTGAAGGCCG | GCAATCTTCATCCTCCTCCAGG |
| R2 | CAGAGCTTTGGTCAGGAAGTG | CGGCCTTCAGGCTTTATGC |
| R3 | TGCCGAGTACAGAGCACAC | CCTGTCCTCTGTGCATCTGC |
| **GFAP** |  |  |
| R1 | CTGATTTTCACTGCCCAGCAC | CCTGACTTCCTGGAAGCACC |
| R2 | TCCTGAGAGAATGAGGGGTACC | GCAGACTTCCTGGCTCTCAC |
| R3 | GATGTGGCTGGAGCCTAAGG | CGCCTTAGCCATCTGTGTCC |
