# Supplementary material for: IFN-γ-STAT1-mediated CD8+ T-cell-neural stem cell cross talk controls astrogliogenesis after spinal cord injury
Source: Inflamm Regen. 2023 Feb 13;43:12. doi: 10.1186/s41232-023-00263-9 (PMC9926765; doi:10.1186/s41232-023-00263-9)
Supplement: Supplementary file 2 — Additional file 2: Supplementary Figure 1. Infiltration of increasing activated CD8+ T cells after SCI. Supplementary Figure 2. Flow cytometry analysis showed the efficiency of transplantation or depletion (B) of CD8+ T cells in spinal cord of SCI mice. Supplementary Figure 3. Identification and differentiation of cultured NSCs. Supplementary Figure 4. ELISA showed the expression of IFN-γ in conditioned medium. Supplementary Figure 5. Western blotting showed the expression of Stat1 in NSCs with or without Stat1 siRNA. Supplementary Figure 6. CD8+ T cells inhibited the differentiation of NSCs into oligodendrocytes and neurons. Supplementary Figure 7. Depleting CD8+ T cells promotes white matter repair after SCI. [file 41232_2023_263_MOESM2_ESM.pdf]

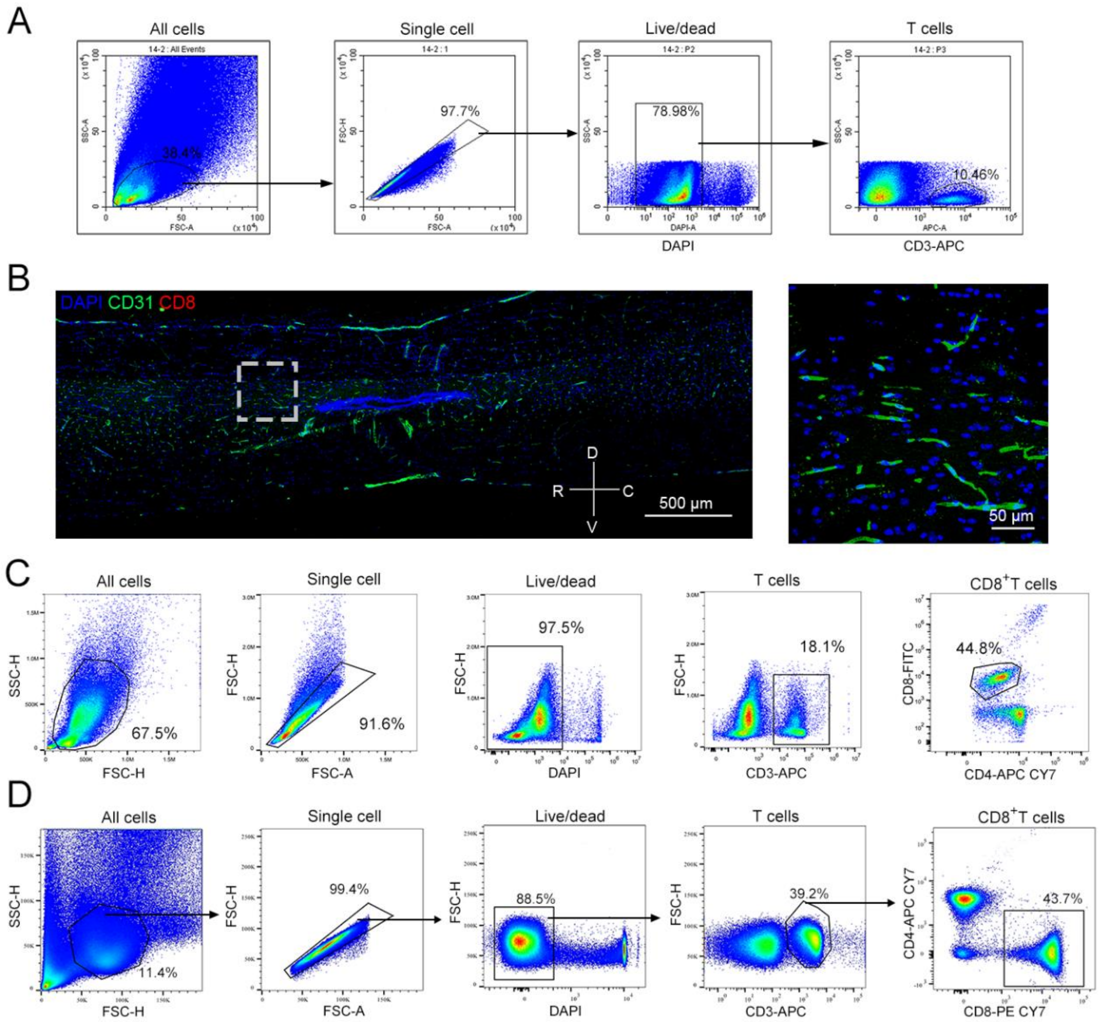

Supplementary Figure 1. Infiltration of increasing activated CD8$^+$ T cells after SCI. (**A**) Gating strategy for total T cells (CD3$^+$), which are used for further identification of CD8$^+$ T cells (CD3$^+$CD4$^-$CD8$^+$) shown in Fig.1A (**B**) Immunofluorescence image of a sagittal section showing no CD8$^+$ T cells (red) in healthy spinal cord. CD31 (green) is used to label endothelial cells of blood vessels. V, ventral; D, dorsal; R, rostral; C, caudal. Scale bar: 500 μm or 50 μm. (**C**) Gating strategy for CD8$^+$ T cells, which are used for further analysis of CD69 expression shown in Fig.1E. (**D**) Gating strategy for CD8$^+$ T cells, which are used for further analysis of CTL-4 and PD-1 expression shown inFig.1F-G.

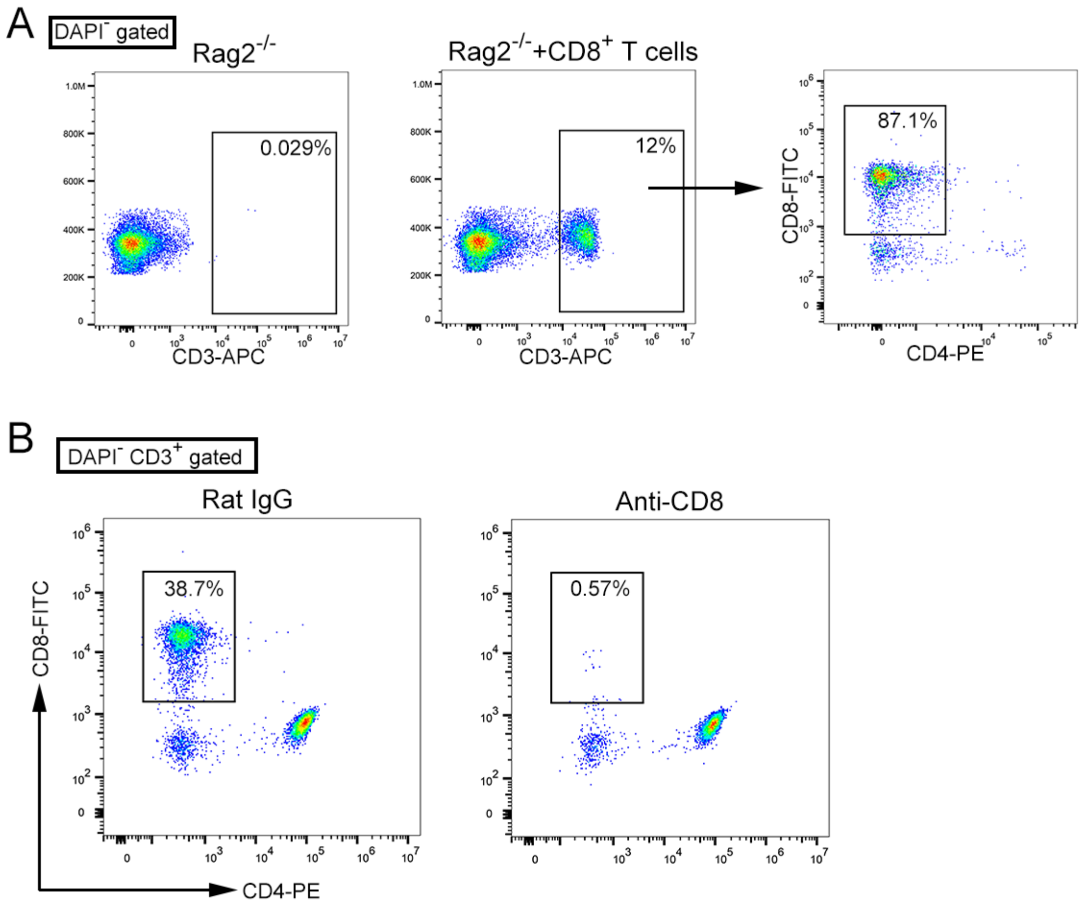

Supplementary Figure 2. Flow cytometry analysis showed the efficiency of transplantation or depletion (B) of CD8$^+$ T cells in spinal cord of SCI mice. (**A**) Flow cytometry showing that few CD3$^+$ T cells were found in Rag2$^{-/-}$ mice, whereas the percentage of CD3$^+$ T cells (87% of which were CD3$^+$CD4$^-$CD8$^+$ T cells) increased in Rag2$^{-/-}$ mice after the adoptive transfer of CD8$^+$ T cells. (**B**) Flow cytometry showing that the anti-CD8 antibody successfully exhaust CD8$^+$ T cells in wide type mice.

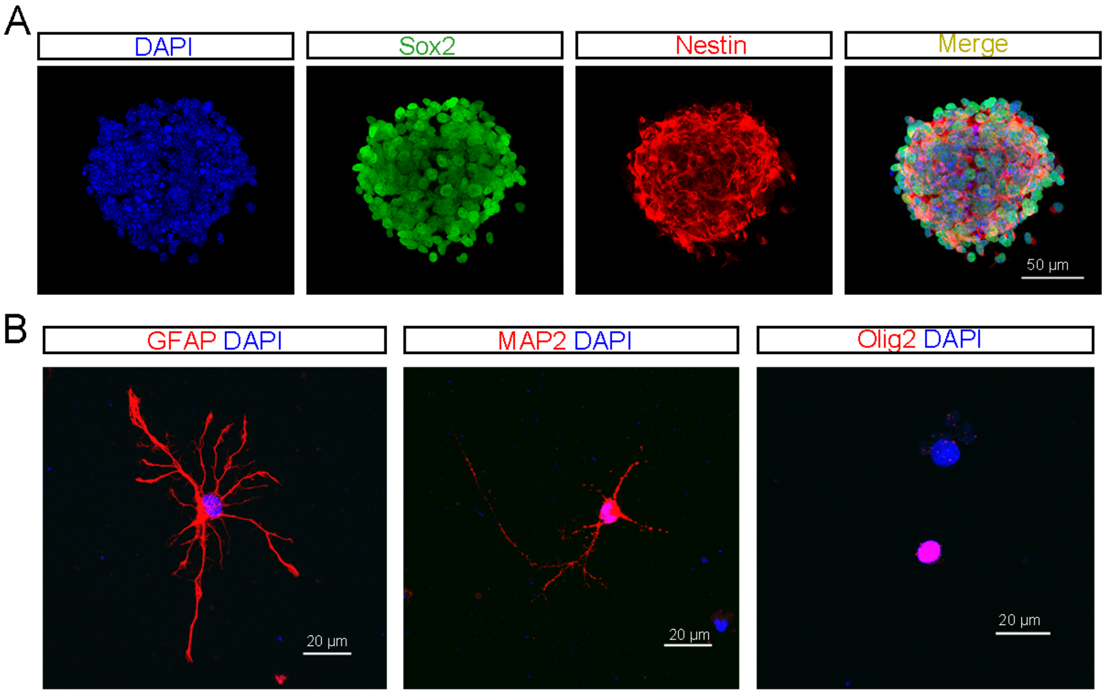

Supplementary Figure 3. Identification and differentiation of cultured NSCs. (**A**) IF staining showed the expression of NSC markers (Nestin and Sox2) in cultured NSCs. Scar bar: 50 μM. (**B**) Immunofluorescence staining showed that cultured NSCs can differentiate into GFAP^{+} astrocytes, MAP2^{+} neurons, and Olig2^{+} oligodendrocytes. Scar bar: 20 μM.

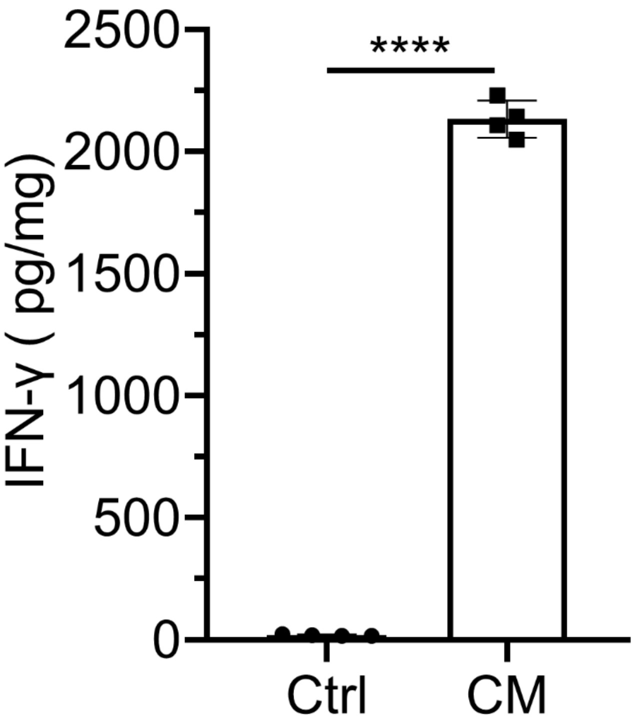

Supplementary Figure 4. ELISA showed the expression of IFN-γ in conditioned medium. N = 4, ****P < 0.0001, Student's t test.

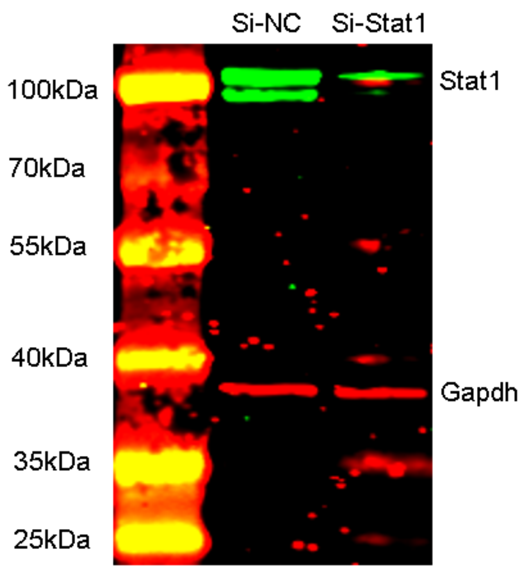

Supplementary Figure 5. Western blotting showed the expression of Stat1 in NSCs with or without Stat1 siRNA.

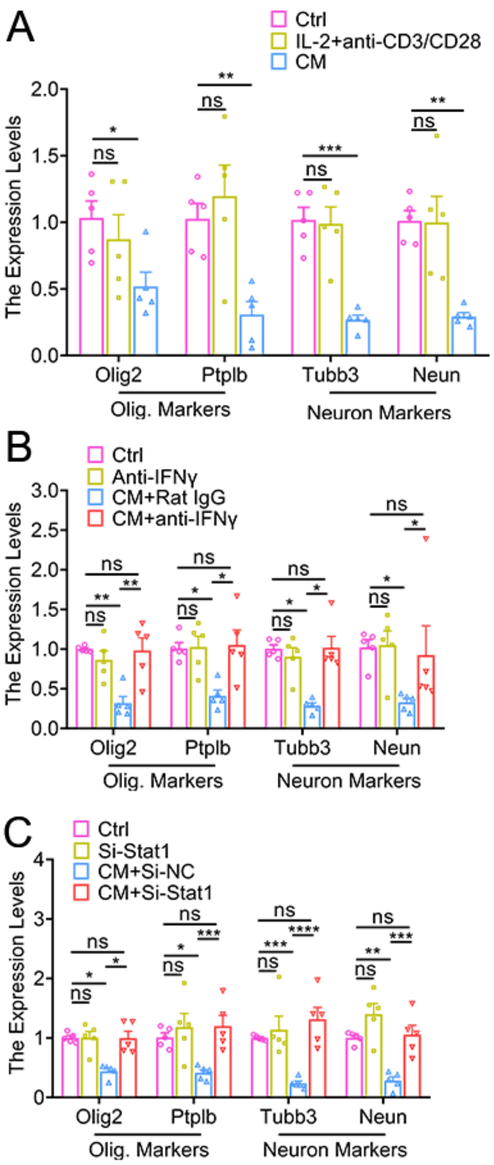

Supplementary Figure 6. CD8$^+$ T cells inhibited the differentiation of NSCs into oligodendrocytes and neurons. (A) qRT-PCR showing the expression of oligodendrocyte markers (Olig2 and Ptplb) and neuron markers (Tubb3 and Neun) in NSCs with or without a conditioned medium for 7 days. (B) qRT-PCR showing the expression levels of Olig2, Ptplb, Ptplb, and Neun in NSCs with or without an anti-IFN-γ antibody for 7 days. (C) qRT-PCR showing the expression levels of Olig2, Ptplb, Ptplb, and Neun in NSCs with or without Stat1 siRNA for 7 days. n = 5, *P < 0.05, **P < 0.01, ****P < 0.0001, one-way ANOVA with Tukey.

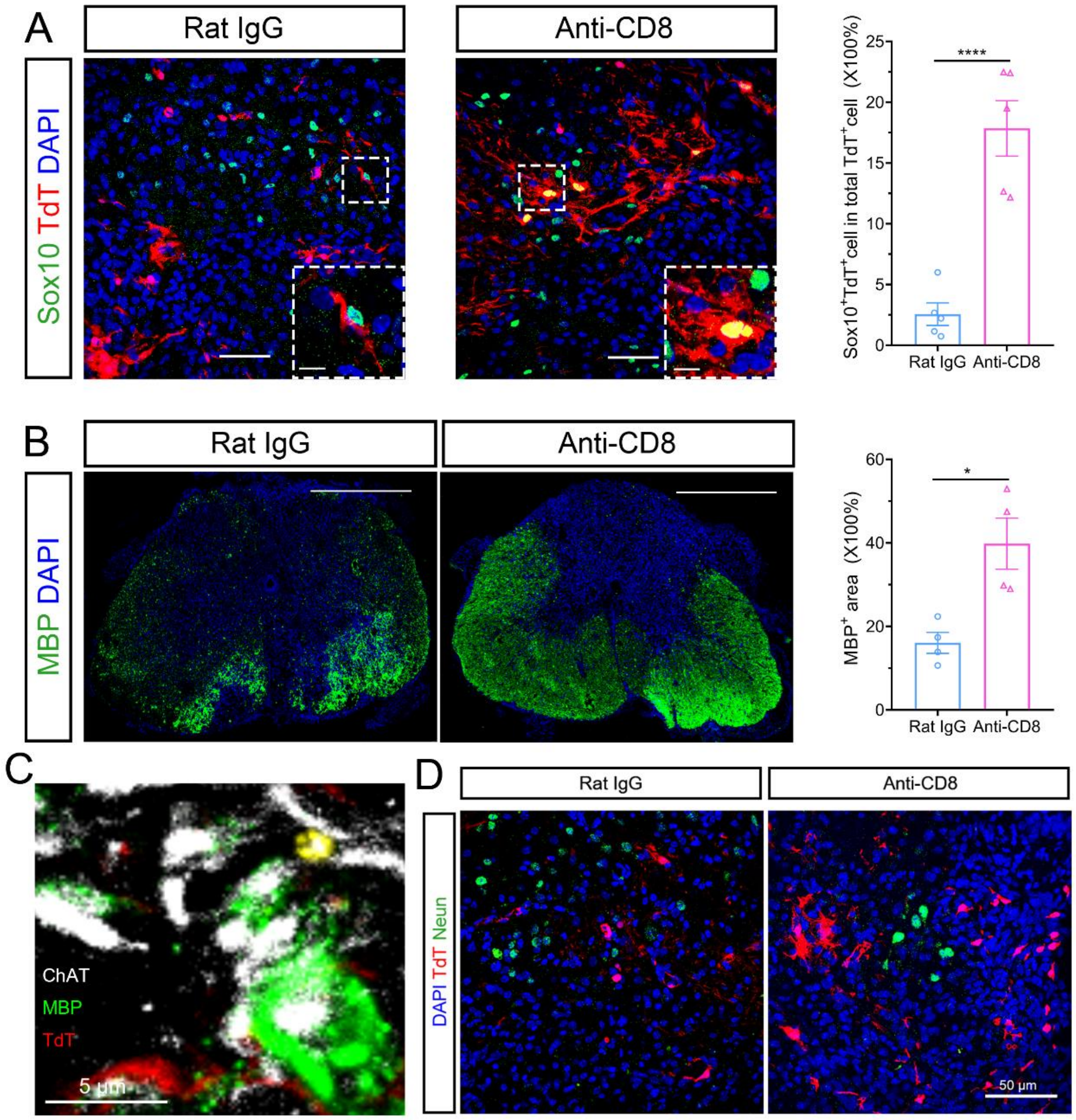

Supplementary Figure 7. Depleting CD8$^+$ T cells promotes white matter repair after SCI. (**A**) IF staining showing the percentage of Sox10$^+$ oligodendrocytes differentiated from NSCs in lineage tracing mice after the administration of an anti-CD8 antibody at 14 dpi. Equal Rat IgG was used for control, n = 5, ****P < 0.0001, Student's t test. Scale bar: 50 μm or 10 μm. (**B**) IF staining showing the area of MBP$^+$ myelin sheath after the administration of an anti-CD8 antibody at 35 dpi. Equal Rat IgG was used for control, n = 4, *P < 0.05, Student's t test. Scale bar: 500 μm. (**C**) IF staining showing that ChAT$^+$ motor neuronal axon can be surrounded by NSC-derived myelin sheath (TdT$^+$, MBP$^+$) after depleting CD8$^+$ T cells at 21 dpi. Scale bar: 5 μm. (**D**) IF staining showing that neuron cannot be differentiated from NSCs after SCI with or without depleting CD8$^+$ T cells. Scale bar: 50 μm.
